# Future Cardiovascular Disease Risk for Women With Gestational Hypertension: A Systematic Review and Meta‐Analysis

**DOI:** 10.1161/JAHA.119.013991

**Published:** 2020-06-24

**Authors:** Charmaine Chu Wen Lo, Andre C. Q. Lo, Shu Hui Leow, Grace Fisher, Beth Corker, Olivia Batho, Bethan Morris, Monika Chowaniec, Catherine J. Vladutiu, Abigail Fraser, Clare Oliver‐Williams

**Affiliations:** ^1^ Faculty of Health and Medicine University of Newcastle New South Wales Australia; ^2^ Liverpool Hospital Liverpool New South Wales Australia; ^3^ Homerton College University of Cambridge United Kingdom; ^4^ Hills Road Sixth Form College Cambridge United Kingdom; ^5^ Department of Obstetrics & Gynecology School of Medicine University of North Carolina Chapel Hill NC; ^6^ Population Health Sciences Bristol Medical School University of Bristol United Kingdom; ^7^ Cardiovascular Epidemiology Unit Department of Public Health and Primary Care University of Cambridge United Kingdom

**Keywords:** cardiovascular disease, gestational hypertension, pregnancy, review, women, Epidemiology, Pregnancy, Women, Risk Factors, Hypertension

## Abstract

**Background:**

Inconsistent findings have been found among studies evaluating the risk of cardiovascular disease for women who have had pregnancies complicated by gestational hypertension (the new onset of high blood pressure without proteinuria during pregnancy). We provide a comprehensive review of studies to quantify the association between gestational hypertension and cardiovascular events in women.

**Methods and Results:**

We conducted a systematic search of PubMed, Embase, and Web of Science in March 2019 for studies examining the association between gestational hypertension and any cardiovascular event. Two reviewers independently assessed the abstracts and full‐text articles. Study characteristics and the relative risk (RR) of cardiovascular events associated with gestational hypertension were extracted from the eligible studies. Where appropriate, the estimates were pooled with inverse variance weighted random‐effects meta‐analysis. A total of 21 studies involving 3 60 1192 women (127 913 with gestational hypertension) were identified. Gestational hypertension in the first pregnancy was associated with a greater risk of overall cardiovascular disease (RR, 1.45; 95% CI, 1.17–1.80) and coronary heart disease (RR, 1.46; 95% CI, 1.23–1.73), but not stroke (RR, 1.26; 95% CI, 0.96–1.65) or thromboembolic events (RR, 0.88; 95% CI, 0.73–1.07). Women with 1 or more pregnancies affected by gestational hypertension were at greater risk of cardiovascular disease (RR, 1.81; 95% CI, 1.42–2.31), coronary heart disease (RR, 1.83; 95% CI, 1.33–2.51), and heart failure (RR, 1.77; 95% CI, 1.47–2.13), but not stroke (RR, 1.50; 95% CI, 0.75–2.99).

**Conclusions:**

Gestational hypertension is associated with a greater risk of overall cardiovascular disease, coronary heart disease, and heart failure. More research is needed to assess the presence of a dose–response relationship between gestational hypertension and subsequent cardiovascular disease.

**Registration:**

URL: https://www.crd.york.ac.uk/prosp​ero/; Unique identifier: CRD42018119031.

**Nonstandard Abbreviations and Acronyms**
ARI: absolute risk increases
CHD: coronary heart disease
CVD: cardiovascular disease
GH: gestational hypertension
HR: hazard ratio
*ICD*: *International Classification of Diseases*
IRR: incident rate ratio
MI: myocardial infarction
OR: odds ratio
RR: relative risk

**Clinical Perspective**

**What Is New?**

- In a systematic review of >3 million women, we found that gestational hypertension is associated with a greater risk of cardiovascular disease, coronary heart disease, and heart failure.
- Nonsignificant trends toward a greater risk of stroke after gestational hypertension were found.

**What Are the Clinical Implications?**

- Women with a pregnancy complicated by gestational hypertension are at greater risk of developing several different kinds of cardiovascular disease.
- Women who experience gestational hypertension may benefit from counseling during and/or after pregnancy about their long‐term cardiovascular risk.

Gestational hypertension (GH), also known as pregnancy‐induced hypertension, is defined as the onset of high blood pressure (at least 140 mm Hg systolic or 90 mm Hg diastolic) without proteinuria on 2 occasions at least 4 hours apart in an ordinarily normotensive pregnant woman after 20 weeks of gestation.^1^, ^2^ Rates of GH vary between countries, with 1% to 6% of pregnancies complicated by GH in Western countries.^3^, ^4^

Pregnancy‐induced hypertension is increasingly recognized as a risk factor for subsequent cardiovascular disease (CVD) in women.^5^ In particular, pre‐eclampsia, characterized by GH with proteinuria, is associated with a markedly higher CVD risk^6^, ^7^, ^8^ and has been incorporated in the American Heart Association guidelines for the assessment of CVD risk in women.^9^ It is unclear if GH and pre‐eclampsia are manifestations of different severities of the same pathophysiological mechanism or represent separate pathologies.^10^ Therefore, the raised CVD risk in women with a history of pre‐eclampsia may not be representative of the risk associated with GH.

Studies that have assessed the CVD risk associated with GH have found mixed results. Results have ranged from no raised risk^11^, ^12^, ^13^ to more than twice the risk of some cardiovascular events.^13^, ^14^, ^15^, ^16^, ^17^, ^18^ This lack of clarity about the long‐term cardiovascular risk for women who have had GH without proteinuria is further underscored by calls for further research into this area by the UK's National Institute for Health and Care Excellence.^19^ Consequently, we conducted a systematic review and meta‐analysis of prospective studies to evaluate the risk of a range of cardiovascular events for women after 1 or more pregnancies complicated by GH.

## METHODS

The design, implementation, analysis, and reporting for this systematic review and meta‐analysis are in accordance with the Meta‐Analysis of Observational Studies in Epidemiology^20^ and Preferred Reporting Items for Systematic Reviews and Meta‐Analyses^21^ protocols (Tables S1 and S2). An internal study protocol was developed to perform this review, which is registered on PROSPERO (https://www.crd.york.ac.uk/prosp​ero/; review reference number CRD42018119031).^22^ The authors declare that all supporting data are available within the article and its online supplementary files.

### Search Strategy and Selection Criteria

We searched the databases PubMed, Embase, and Web of Science in March 2019. No restrictions were applied to the language or publication period of the articles. Both medical search headings and open‐text fields were used to identify articles.

The exposure was GH and any cardiovascular outcome was of interest, including (1) overall CVD; (2) coronary heart disease (CHD); (3) any stroke, including ischemic and hemorrhagic stroke; (4) heart failure; and (5) thromboembolic events. The details of the search terms are provided in Table S3. The search in PubMed was restricted to articles relating to humans. We cross‐referenced the bibliographies of any relevant journal articles and systematic reviews we identified during our search to determine if there were any additional studies not found in our original search that fit our inclusion criteria.

To be included in the review, the articles had to compare the risk of at least 1 cardiovascular outcome for women with previous GH with that of women who had 1 or more normotensive pregnancies. GH was defined as a new onset of systolic and/or diastolic hypertension after 20 weeks gestation without proteinuria. Events had to occur more than 1‐year postpartum to minimize the risk of comorbidity. Articles only evaluating pre‐eclampsia, or combining both pre‐eclampsia and GH as an exposure, were excluded to minimize heterogeneity in the exposure. Study designs were limited to cohort studies and case‐control studies. Exclusion criteria were the following: (1) studies that included animals, men, children, or nulliparous women; (2) studies that did not have a cardiovascular outcome; (3) studies that combined women with GH and women with pre‐eclampsia; and (4) studies that did not evaluate GH as an independent exposure.

### Selection of Studies and Data Extraction

Using the software Abstrackr,^23^ each abstract found with our search strategy were screened by 2 authors (C.C.W.L., A.C.Q.L., S.H.L., G.F., B.C., O.B., B.M., or M.C.). Any differences between reviewers were discussed and resolved by a third individual (C.O.‐W.). For relevant abstracts, full texts were accessed to determine their eligibility for the review. Where 2 studies evaluated the same outcome in the same cohort, the study with the longer follow‐up time was used. Data on the follow‐up period, study design, population characteristics, sample size, exposure and outcome, methods of ascertainment for GH and cardiovascular events, and adjustment factors were abstracted and independently verified by a second author. Both minimally adjusted and fully adjusted measures of the association and 95% CIs were also extracted and verified. Any differences between reviewers were discussed and resolved by a third author.

For the fully adjusted measures of association, studies were categorized as poorly, adequately, or well adjusted. To be considered well adjusted, studies had to control for maternal age; socioeconomic factors; obstetric history, including pregnancy complications other than GH; and chronic diseases. We selected these categories as they broadly cover most potential confounders and are representative of the range of adjustments made in the studies included in the review. Adequately adjusted studies controlled for variables from 3 of these 4 categories, and poorly adjusted studies controlled for variables in 2 or fewer categories.

Two authors independently evaluated the bias within each individual study using the validated Newcastle–Ottawa Scale, a semiquantitative scale designed to evaluate the quality of nonrandomized studies.^24^ It allocates a maximum of 9 stars to a study. Study quality was judged on the selection criteria of participants, comparability of groups through adjustment, and exposure or outcome assessment.

### Statistical Analysis

The included studies used 2 different approaches to classify GH exposure. The first approach classified women based on the presence or absence of a diagnosis of GH in the first pregnancy. The second approach classified women as having either a history of 1 or more pregnancies affected by GH or only having normotensive pregnancies. Because of the distinction between these 2 classifications, our meta‐analyses were conducted assessing risk associated with 2 exposures: (1) a diagnosis of GH in the first pregnancy and (2) a history of 1 or more pregnancies affected by GH.

For a meta‐analysis to be conducted, it was necessary to identify a minimum of 3 studies evaluating the risk of a particular cardiovascular outcome (eg, stroke, CHD) associated with 1 of these exposures. If fewer than 3 studies were found for an exposure–outcome combination, then the results were included in the systematic literature review, but not in the meta‐analysis.

For studies that reported separate relative risk (RR) estimates for subgroups (eg ethnic groups) or that reported CHD and overall stroke risk estimates separately for the same population, but did not report an overall CVD risk estimate, we used inverse variance weighted fixed effects meta‐analysis to generate overall study‐level RRs before combining these results with those from other studies.

When pooling the results from separate studies, the inverse variance weighted method was used to combine odds ratio (OR), RR, and hazard ratios (HR) to produce a pooled RR under the rare outcome assumption. Random effects analyses using the DerSimonian–Laird model were used to allow for between‐study heterogeneity as there were clear differences between the identified studies, such as ethnicity. Heterogeneity was assessed using the Cochrane χ^2^ statistic and the I^2^ statistic. Individual RR estimates and summary estimates were displayed graphically with forest plots.

To assess the number of cases that could be avoided if effective intervention for CVD are targeted to women with GH, the absolute risk increases (ARI) for overall CVD and CHD were calculated separately for both exposures. The equation ARI=(RR−1)×(assumed control risk) was used, where RR is from the meta‐analysis.

Female‐specific European Heart Network statistics for 2015 were used to estimate the assumed control risk (ie, the incidence) of overall CVD and CHD because the largest number of studies came from Europe.^25^ ARI were expressed as events per 1000 woman‐years of follow‐up. It was not possible to calculate the ARI for heart failure or thromboembolic events as we could not obtain estimates of their incidence. The ARI was not calculated for stroke because of the nonsignificant results in the main meta‐analyses.

### Sensitivity Analyses

A number of sensitivity analyses were conducted. The first analysis excluded studies with the largest effect estimates to assess the impact of these studies on the magnitude of the pooled result and the observed heterogeneity. The second analysis included all studies and reran all meta‐analyses with fixed effects models. This was performed because the DerSimonian–Laird method for random effects meta‐analysis may have statistical limitations in the case of few studies.^26^ Therefore a fixed effects meta‐analysis will provide an assessment of the consistency of the results and an estimation of the relationships specifically in the overall populations studied. Several studies assessed the risk of stroke subtypes (intracerebral hemorrhage and ischemic stroke) associated with a history of GH. To assess the risk of any stroke outcome, an additional meta‐analysis was conducted that combined risk estimates for overall stroke and stroke subtypes associated with a history of GH.

A total of 5 stratified analyses were conducted to evaluate (1) the effect of different levels of adjustment, (2) the potential impact of bias in individual studies, and (3) the effect of study‐level characteristics on the association between GH and overall CVD. Only overall CVD was assessed as an outcome because too few studies were included in the meta‐analyses of other events. Analyses were stratified by (1) level of adjustment, (2) risk of bias, (3) duration of follow‐up, (4) year of publication, and (5) the population studied. In these analyses, we tested for trend across strata using random effects meta‐regression.

Small study effects were evaluated through funnel plots and Egger tests for meta‐analyses including 6 or more studies.^27^ Upon evidence of funnel plot asymmetry and indication of significant bias from the Egger test, the trim‐and‐fill method was used to correct for funnel plot asymmetry.^28^

All tests were 2‐tailed and *P* values of <0.05 were considered statistically significant. STATA software package (version 14.2; Stata Corp, College Station, TX) was used for all statistical analyses.

## RESULTS

**Figure 1:**
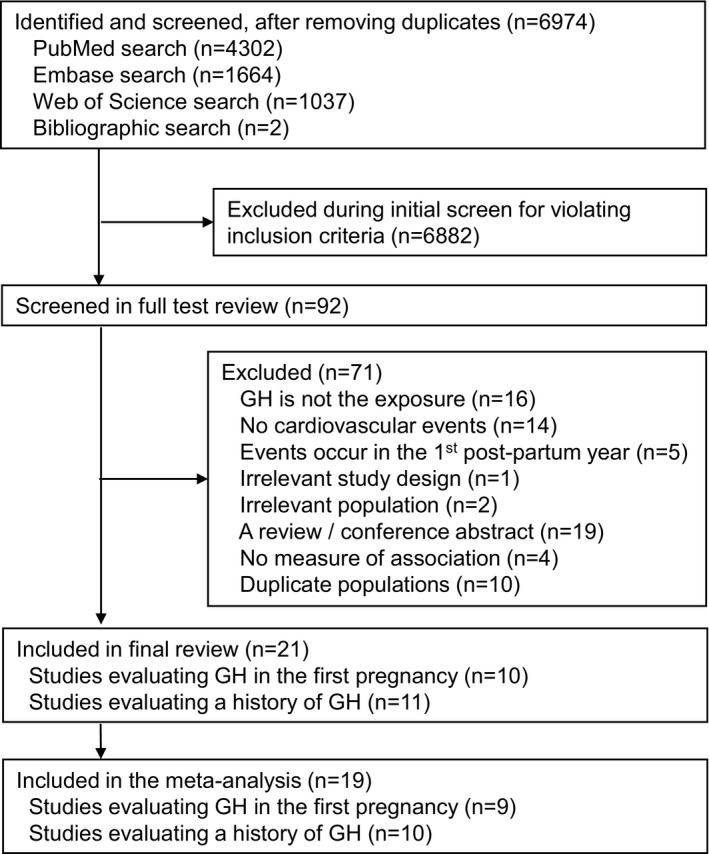
Identification of studies included in the review of GH and risk of cardiovascular events. GH indicates gestational hypertension.

Our search strategy identified 6974 studies, of which 6882 were excluded during the initial abstract screen. The remaining 92 articles were reviewed in full, resulting in 71 being excluded and 21 included in our final review (Figure 1). The studies included 3 601 192 women, with 127 913 women with a history of 1 or more pregnancies affected by gestational hypertension from 18 cohort studies^11^, ^12^, ^13^, ^29^, ^30^, ^31^, ^32^, ^33^, ^34^, ^35^, ^36^, ^37^, ^38^, ^39^ and 3 nested case‐control studies.^15^, ^18^, ^40^ Studies were conducted in Europe (12 studies^*^) and North America (5 studies^15^, ^17^, ^31^, ^32^, ^36^) as well as in Taiwan (2 studies^18^, ^41^) and Australia (1 study^13^) (Table).

**Table 1:** Characteristics of Studies Included in the Review

First Author, y	Details of Cohort	Study Design	No. of Women	No. of Women With GH	GH Definition	Method of GH Ascertainment	Duration of Follow‐Up,y	Age at Enrollment, y	Outcome(s)	Method of Outcome Ascertainment
Andolf et al 2017^29^	Swedish National Register Study 1973–2009	Cohort study	283 990	4762	*ICD* codes: ICD‐8	Medical records	Mean: 35	Mean: 26.19	Heart failure^a^	Medical records
Behrens et al 2016^30^	Danish medical registries, 1978–2012	Cohort study	834 919	11 047	*ICD* codes: ICD‐8, ICD‐10	Medical records	Mean: 17.9	Median: 25–29	Cardiomyopathy	Medical records
Bhattacharya et al 2012^11^	Aberdeen Maternity and Neonatal Databank and NHS medical records, 1950–2008	Cohort study	32 828	8891	Diastolic pressure >90 mmHg on two occasions at least four hours apart or one reading of >110 mmHg	Medical records	Max: 58	Mean: 24.27	CVD, CHD, stroke, pulmonary embolism	Medical records
Cain et al 2016^31^	Florida maternal and infant databases, 1998–2009	Cohort study	302 686	17 150^b^	*ICD* codes ICD‐9‐CM	Medical records	Median: 4.9	Mean: 25.1	CVD	Medical records
Cirillo et al 2015^32^	US Child Health and Development Studies, 1959–2011	Cohort study	10 721	1662	≥1 blood pressure reading of >140/90 mm Hg after 20 wk gestation	Medical records	Range: 44–52	Median: 26	Fatal CVD	Death certificates
Grandi et al 2017^b^, ^14^	UK Clinical Database, 1990–2013	Cohort study	146 000	Not given	Read codes	Medical records	Median: 4.7	Mean: 29.24	CVD	Medical records
Kestenbaum et al 2003^15^	Washington State Birth Events Record Database & Comprehensive Hospital Abstract Reporting System database, 1987–2001	Nested Case Control	103 589	10 687	*ICD* codes: ICD‐9‐CM	Birth certificate data	Mean 7.8	Mean: 26.23	CVD, thromboembolic events	Medical records
Lin et al 2016^41^	Taiwan National Health Insurance Database, 2000–2013	Cohort study	36 950	7390	*ICD* codes: ICD‐9‐CM	Health insurance claims data	Max: 13	Mean: 31.06	Intracerebral hemorrhage	Health insurance claims data
Luoto et al 2008^12^	Women giving birth in Helsinki hospitals, 1954–2005	Cohort study	4000	98	Coding not specified	Medical records	Mean: 44	Not given	Fatal CVD	Medical records
Lykke et al 2009^34^	Danish medical registries, 1978–2007	Cohort study	782 287	7449	*ICD* codes: ICD‐8, ICD‐10	Medical records	Mean: 14.6	Mean: 26.8	CHD, heart failure, thromboembolic event, stroke	Medical records
Lykke et al 2010^33^	Danish medical registries, 1978–2007	Cohort study	782 287	7449	*ICD* codes: ICD‐8, ICD‐10	Medical records	Median: 14.8	Mean: 26.8	Fatal CVD	Medical records
Männistö et al 2013^35^	Northern Finland Birth Cohort, 1966–2000	Cohort study	7543	991	SBP ≥145 mm Hg and/or DBP ≥95 mm Hg	Assessed during pregnancy as part of study	Mean: 39.4	Mean: 26.76	CHD, MI, heart failure, stroke	Medical records
Ray et al 2005^36^	Ontario Health Insurance Plan, 1990–2004	Cohort study	963 263	20 942	*ICD* codes: ICD9	Healthcare administrative databases	Median 8.7	Mean: 28	CVD	Hospital database
Riise et al 2018^37^	Norweigian registries, 1980–2009	Cohort study	587 755	11 600	SBP ≥140 mm Hg, DBP ≥90 mm Hg, or >15 mm Hg BP increase measured <20 wk gestation	Medical records	Median: 14.3	Mean: 26.3	CVD, CHD, stroke	Medical records
Riise et al 2019^38^	Norweigian registries, 1980–2009	Cohort study	20 075	364	SBP ≥140 mm Hg, DBP ≥90 mm Hg, or >15 mm Hg BP increase measured <20 wk gestation	Medical Records	Median: 11.4	Mean: 26.0	Composite: acute myocardial infarction or acute cerebral stroke	Medical records
Schmiegelow et al 2014^16^	Danish registries, 2004–2009	Cohort study	273 101	2903	*ICD* codes: ICD‐8, ICD‐10	Medical records	Median: 4.5	Median: 30.4	MI, ischemic stroke, CVD	Medical records
Theilen et al 2016^17^	Utah Population Database, 1939–2012	Cohort study	152 034	28 894	Coding not specified	Birth certificates	Max: 73	Mean: 26.0	CHD, stroke	Medical records
Tooher et al 2017^13^	Royal Prince Alfred Women and Babies hospital, Australia, 1980–2009 onward	Cohort study	27 887	625	*ICD* codes: ICD‐9‐AM	Medical records	Median: 20‡	Mean: 27	CVD, CHD, stroke	Registry, discharge
Wikstrom et al 2005^39^	Swedish Medical Birth Register, 1987–2001	Cohort study	391 017	7936	*ICD* codes: ICD‐8	Medical records	Max: 15	Range: 15–64	CHD	Registry (cause of death, hospital discharge)
Wilson et al 2003^40^	Aberdeen Maternity and Neonatal Databank, 1951–1999	Nested case control	2394	1197	DBP ≥90 mm Hg twice at 4+ h apart or 1 reading of ≥110 mm Hg	Medical records	Max: 48	Mean: 24.2	Angina, MI, DVT, other circulatory disease (not hypertension, CHD or cerebrovascular disease)	Medical and death records
Yeh et al 2014^18^	Taiwan National Health Insurance database, 1998–2009	Nested case‐control	5765	725	*ICD* codes: ICD‐9‐CM	Health insurance claims data	Median: 5.8	Mean: 29.8	CVD	Medical records

CHD indicates coronary heart disease; CVD, cardiovascular disease; DBP, diastolic blood pressure; DVT, deep vein thrombosis; GH, gestational hypertension; *ICD*,* International Classification of Diseases*; MI, myocardial infarction; NHS, National Health Service; and SBP, systolic blood pressure.

a Stroke, CHD, and CVD also reported, but not included in the meta‐analysis as the same population used in Lykke et al.^34^

b Cain et al^31^ and Grandi et al^14^ did not indicate how many patients had GH, and the total number of women was estimated.

Median time from index pregnancy to onset of CVD—no follow‐up duration given for full cohort.

All of the studies ascertained GH and cardiovascular events through medical records, registry data, or health insurance claims (Table, Table S4). The duration of follow‐up varied from a median of 4.5 years^16^ to a maximum of 73 years^17^ (Table). Based on the Newcastle–Ottawa scale, 5 studies were judged to be at high risk of bias, and 10 studies provided risk estimates that were poorly adjusted (Tables S5 and S6).

### GH in the First Pregnancy

**Figure 2:**
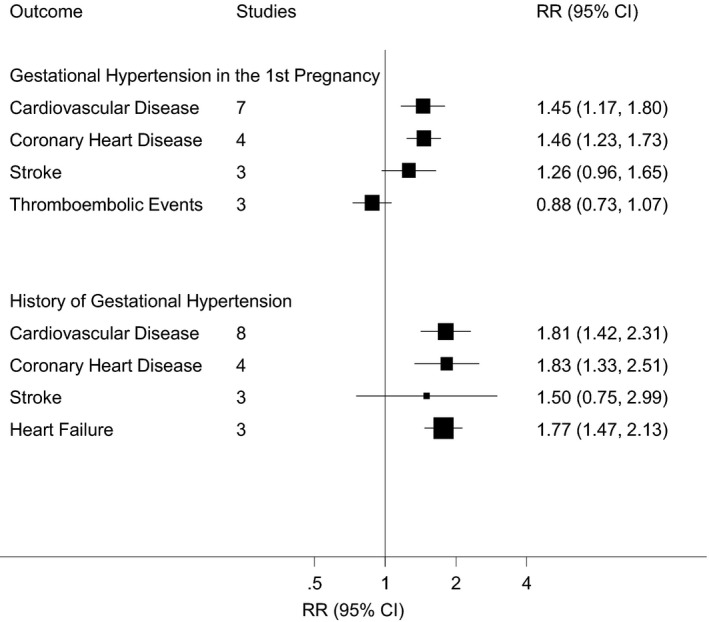
Association between gestational hypertension and cardiovascular events, showing summary RRs for the meta‐analyses of each outcome. RR indicates relative risk.

A total of 11 studies,^11^, ^12^, ^14^, ^31^, ^33^, ^34^, ^36^, ^37^, ^38^, ^39^, ^40^ including 3 209 836 women (74 066 with GH), examined the risk of cardiovascular events in women whose first pregnancy was affected by GH. The risk of the following events was assessed: overall CVD, CHD, heart failure, any stroke, myocardial infarction (MI), thromboembolic events, angina, other circulatory disease, and a combined outcome of acute MI and acute cerebral stroke (Figure 2, Tables S7 and S8). Of the 9 included cohorts, GH affected 1.0% to 27.1% of first pregnancies. Meta‐analyses included 2 818 819 women (66 130 with GH) for overall CVD, 1 793 887 women (35 876 with GH) for CHD, 1 402 870 women (27 940 with GH) for stroke, and 1 402 870 women (27 940 with GH) for thromboembolic events.

**Figure 3:**
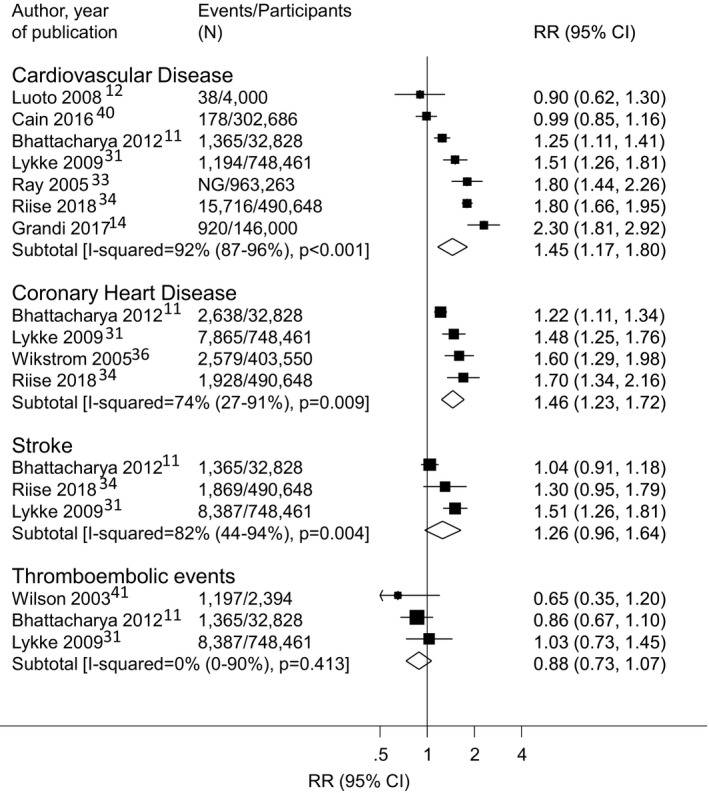
Association between gestational hypertension in a woman's first pregnancy and subsequent risk of cardiovascular events in adjusted analyses. RR indicates relative risk.

Meta‐analyses of adjusted estimates found a significantly greater risk of overall CVD (7 studies^11^, ^12^, ^14^, ^31^, ^34^, ^36^, ^37^ RR, 1.45; 95% CI, 1.17–1.80) and CHD (4 studies^11,34,37,39^; RR, 1.46; 95% CI, 1.23–1.72), but not overall stroke (3 studies^11^, ^34^, ^37^ RR, 1.26; 95% CI, 0.96–1.64) or thromboembolic events (3 studies^11^, ^34^, ^40^ RR, 0.88; 95% CI, 0.73–1.07) (Figure 3). There was evidence of significant between‐study heterogeneity for overall CVD (I^2^=92%, *P*<0.001), CHD (74%, *P*=0.009), and overall stroke (82%, *P*=0.004), but not thromboembolic events (0%, *P*=0.413). Meta‐analyses of the unadjusted results were consistent with these findings (Figure S1).

The ARI in overall CVD and CHD associated with GH in the first pregnancy, based on the European population, were 8.6 and 4.2 events per 1000 woman‐years, respectively.

Five findings from 3 studies were not included in the meta‐analyses (Table S8). These studies evaluated heart failure, a composite outcome of MI and acute cerebral stroke, angina, MI, and other circulatory disease. Greater risks of heart failure and combined acute MI and acute cerebral stroke were noted, which both attenuated after adjustment (adjusted HR, 1.37; 95% CI, 0.98–1.93; and adjusted HR, 1.8; 95% CI, 0.8–4.1), respectively.^34^, ^38^ One study found no increased risk of MI (adjusted OR, 0.73; 95% CI, 0.32–1.63) or angina (adjusted OR, 1.02; 95% CI, 0.58–1.81), but noted a greater risk of other circulatory disease, defined as circulatory diseases that did not include hypertension, CHD, or cerebrovascular disease (adjusted incident rate ratio [IRR], 1.51; 95% CI, 1.06–2.14).^40^

### History of GH

A total of 11 studies from 10 populations^†^ assessed the risk of a cardiovascular outcome associated with a history of 1 or more pregnancies affected by GH. They included 2 291 304 women (73 994 with GH). The studies evaluated overall CVD, CHD, heart failure, overall stroke, intracerebral hemorrhage, ischemic stroke, MI, and thromboembolic events (Figure 1, Tables S7 and S8). Of the included studies, 9 were cohort studies in which the prevalence of women with a history of GH ranged from 1.1% to 19.0%. Meta‐analyses included 861 087 women (50 356 with GH) for overall CVD, 471 454 women (35 272 with GH) for CHD, 1 126 452 women (16 800 with GH) for heart failure, and 463 911 women (34 281 with GH) for stroke.

**Figure 4:**
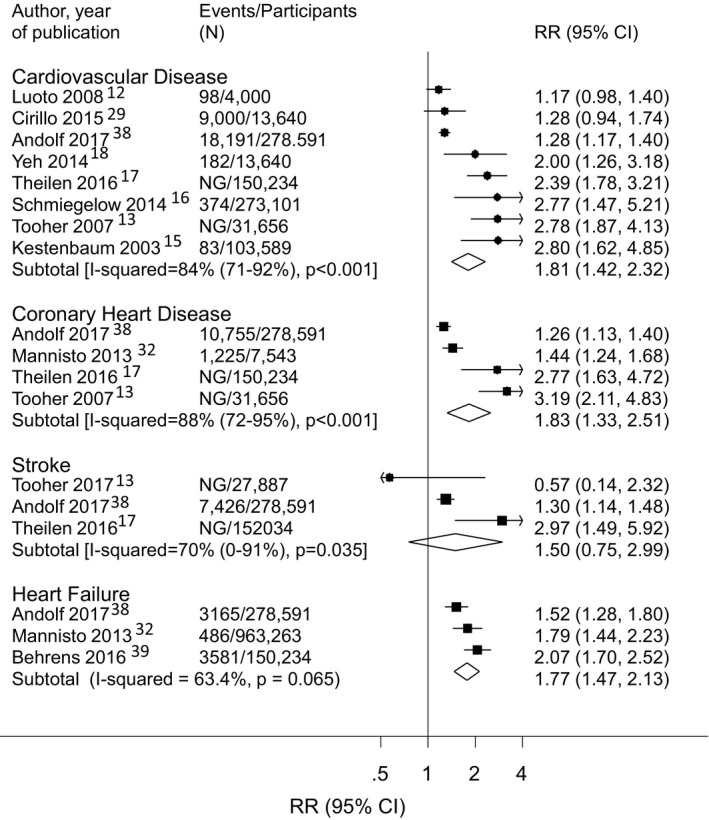
Association between a history of one or more pregnancies affected by gestational hypertension and subsequent risk of cardiovascular events in adjusted analyses. NG indicates not given; and RR, relative risk.

In meta‐analyses of adjusted risk estimates, a history of GH was associated with a greater risk of overall CVD (8 studies^13^, ^15^, ^16^, ^17^, ^18^, ^29^, ^32^ RR, 1.81; 95% CI, 1.42–2.32), CHD (4 studies^13^, ^17^, ^29^, ^35^; RR, 1.83; 95% CI, 1.33–2.51) and heart failure (3 studies^13^, ^17^, ^29^; RR, 1.77; 95% CI, 1.47–2.13), but not overall stroke (3 studies^29^, ^30^, ^35^; RR, 1.50; 95% CI, 0.75–2.99) (Figure 4). There was evidence of high heterogeneity in all analyses: overall CVD (84%, *P*<0.001), CHD (88%, *P*<0.001), heart failure (63%, *P*=0.065), and overall stroke (70%, *P*=0.035). A greater CVD risk was also observed in the meta‐analysis of unadjusted findings (Figure S2).

The ARI in overall CVD and CHD associated with a history of GH, based on the European population, were 15.6 and 7.6 events per 1000 woman‐years, respectively.

Findings from 7 studies were not included in the meta‐analysis (Table S8). These studies evaluated the risk of MI, intracerebral hemorrhage, ischemic stroke, cardiomyopathy, and thromboembolic events. Evidence of higher risks were found for cardiomyopathy (HR, 1.83; 95% CI, 1.20–2.63), intracerebral hemorrhage (IRR, 3.62; 95% CI, 3.63–3.81) and, in 2 studies, ischemic stroke (IRR, 1.59; 95% CI, 1.24–2.04; HR, 2.78; 95% CI, 1.13–6.82).^16^, ^30^, ^35^, ^41^ A history of GH was also associated with MI in 1 study (IRR, 1.75; 95% CI, 1.40–2.19),^35^ but not in a second study (HR, 1.41; 95% CI, 0.19–10.21).^16^ No statistically strong evidence of an association between a history of GH and thromboembolic events was found (HR, 1.5; 95% CI, 0.9–2.5).^15^

Two studies assessed the dose–response relationship between number of pregnancies with GH and a cardiovascular outcome. Both identified cohorts of women with 2 pregnancies who were categorized as having (1) GH in the first pregnancy only, (2) GH in the second pregnancy only, (3) GH in both pregnancies, or (4) GH in neither pregnancy. A greater risk of overall CVD relative to normotensive women was found for women with GH in their first pregnancy (HR, 1.7; 95% CI, 1.5–2.0), their second pregnancy (HR, 2.4; 95% CI, 2.1–2.8), and in both pregnancies (HR, 1.9; 95% CI, 1.8–2.0).^37^ A greater CHD risk was also noted for women with GH in either their first pregnancy (IRR, 1.9; 95% CI, 1.5–2.4) or second pregnancy (IRR, 2.4; 95% CI, 1.8–3.2) and for those with 2 or more affected pregnancies (IRR, 2.8; 95% CI, 2.0–3.9).^39^

### Sensitivity Analyses

Risk estimates were consistent after excluding studies with the largest effect and after conducting a fixed effects meta‐analysis, with I^2^ results staying relatively constant (Table S9). When all stroke events, including overall stroke and stroke subtypes (intracerebral hemorrhage and ischemic stroke), were included in the history of GH meta‐analysis, there was evidence for a greater risk of any stroke outcome for women with 1 or more pregnancies affected by GH: RR, 1.96 (95% CI, 1.06–3.63). Evidence for between‐study heterogeneity was found in this analysis (98%, *P*<0.001) (Figure S3).

The overall CVD analyses were separately stratified by average duration of follow‐up, risk of bias, level of adjustment, year of publication, and population (Table S10). There was no evidence that risk estimates varied between strata, and there remained evidence of heterogeneity in most categories after stratification.

### Small Study Effects

The funnel plot for overall CVD risk after GH in the first pregnancy did not show evidence of asymmetry (Egger test, *P*=0.935) (Figure S4). The funnel plot for a history of GH and overall CVD risk indicated potential asymmetry (*P*=0.051), with publications of small studies with null or negative effect estimates missing (Figure S5). Use of the trim‐and‐fill method resulted in a RR of 1.26 (95% CI, 1.15–1.39). The funnel plot for a history of GH and risk of any stroke outcome did not show evidence of asymmetry (*P*=0.382) (Figure S6).

## DISCUSSION

This systematic review found that women previously diagnosed with GH had a greater risk of overall CVD, CHD, and heart failure and some indication of a greater risk of stroke as well.

This study adds to the literature on the relationship between women's obstetric history and risk of cardiovascular events. A single previous review evaluated cardiovascular events after GH^42^ however, they focused on morbidity from CVD and cerebrovascular disease only. Our findings substantially build on it providing a comprehensive, holistic review of the risk of fatal and nonfatal cardiovascular events after GH.

This study adds to the growing literature on the relationship between women's obstetric history and their subsequent risk of cardiovascular events. These include a greater risk of overall CVD with recurrent miscarriages,^43^ preterm birth,^44^ fetal growth restriction^45^ and pre‐eclampsia.^46^ The magnitude of association for overall CVD risk found in the current review is similar to that found with recurrent miscarriages,^43^ preterm birth^44^ and fetal growth restriction.^45^ Although the overall CVD risk associated with pre‐eclampsia is greater than that of GH.^46^

### Strengths and Weaknesses of the Study

Strengths of this study include the large number of women included and the variety of cardiovascular events assessed, which allowed us to obtain the most holistic picture to date of the effect of GH on long‐term cardiovascular health. Because of the larger number of studies included in the overall CVD analysis, it was possible to assess the impact of study characteristics on the meta‐analysis and to conduct sensitivity analyses. Furthermore, there was sufficient follow‐up duration in many of the studies (10 studies had more than 15 years of follow‐up) for long‐term CVD risk to be adequately assessed. Lastly, diagnoses of GH and cardiovascular events were mainly ascertained through medical records, which reduced possible information bias arising from self‐report.

Nevertheless, our study has limitations. First, it is possible that despite searching multiple databases without language or time restrictions, relevant studies were missed. Second, there were only 21 studies identified, and at most 8 studies were included in any single meta‐analysis, suggesting that analyses could be influenced by a single study. However, exclusion of the studies with the largest effect estimates did not materially alter the conclusions of the meta‐analyses. Few studies were found for some events, such as stroke and thromboembolic events, and thus limited sensitivity analyses.

Third, high heterogeneity (I^2^>70%) was found for most meta‐analyses. This may be attributed to differences in study design, methodology, or population. Stratified analyses in the current review were limited to CVD only and may have been underpowered to detect some of these differences. Other potential sources of heterogeneity include differences in the frequency of postpartum chronic hypertension and variation in outcome and exposure identification. Chronic hypertension is likely to be an important mediator of the relationship between GH and CVD,^40^, ^47^ therefore the frequency of conversion of GH to chronic hypertension may be a source of heterogeneity between populations and thus studies. Outcome definitions may have varied between studies because of the inclusion of different *International Classification of Diseases* (*ICD*) codes to define the same outcome (Table S4). Although all studies used robust measurements of exposure or events through blood pressure measurement and registries, revisions of *ICD* criteria could have led to differences in the definition of *ICD* codes between studies. Furthermore, there are challenges in identifying exposed women as well, as it requires a blood pressure measurement taken before 20 weeks gestation to rule out chronic hypertension, the criteria for which has changed over time, notably in the United States.^48^

Fourth, many studies were of poor quality, and there were different adjustment sets considered, which could have resulted in residual confounding. However, when low‐quality studies were excluded, the results were broadly similar. Fifth, our funnel plot for overall CVD risk with a history of GH indicates some asymmetry where small studies that report a significant, positive result are more likely to be published (Figure S4). Use of the trim‐and‐fill method found that the association would remain after correcting for the asymmetry. Lastly, the majority of studies were from Western populations, which may limit the generalizability of these findings to other populations.

### Implications for Clinical Practice

Several theories have been proposed to explain the link between GH and the development of CVD. Hypertension in pregnancy may cause lasting damage that contributes to CVD. Alternatively, or in addition to this, women who develop GH may have a pre‐existing predisposition to CVD, which unmasks itself during pregnancy. For example, prepregnancy body mass index is particularly important for GH risk^49^ and body mass index, in general, is linked to CVD development.^50^, ^51^ These theories, in combination with the findings of this review, underscore the importance of intervention to decrease CVD risk factors. This could have the dual benefit of decreasing both the severity and incidence of GH and CVD.

The timing of when an intervention is administered merits discussion, and the pathological mechanisms linking GH to CVD development have implications for this. If there is a pre‐existing predisposition to CVD, then intervention before conception should be a priority. There is increasing emphasis on the importance of preconception health and its implications for future health.^52^ However, the challenges of intervening before conception lie in identifying women considering pregnancy and will not aid women with unplanned pregnancies, which may be up to half of all pregnancies in some groups of women.^53^

Intervention during or shortly after pregnancy may be a viable approach and may help mitigate any long‐term damage caused by GH. Strategies for managing cardiovascular risk factors during pregnancy could include lifestyle changes that limit excess gestational weight gain, a known risk factor for GH and other pregnancy complications.^54^, ^55^ There is evidence that lifestyle changes can be effective in mitigating maternal and fetal risks,^56^ and research is underway to identify the ideal interventions.^57^ Women who experience GH may also benefit from counseling during and/or after pregnancy about their long‐term cardiovascular risk. Strategies that could be implemented after pregnancy may include discussion of heart age calculations,^58^, ^59^ which may be more applicable to a younger population of women than predicting their cardiovascular risk, which is likely to be low in the years after giving birth.

### Unanswered Questions and Future Research

Pre‐eclampsia is currently recognized in guidelines for assessing CVD risk in women^9^ however, GH is not. To assess whether GH should also be included in CVD risk guidelines, further research is needed. The risk of some diseases that have been evaluated in relation to GH, such as stroke subtypes, would benefit from further study to confirm the association indicated in this review, whereas many cardiovascular events have been entirely overlooked, such as peripheral arterial disease and transient ischemic attack. Furthermore, only 2 studies were identified that assessed a dose–response relationship, that is, whether the risk of a cardiovascular outcome rises with an increasing number of pregnancies affected by GH. Given the evidence for a dose–response relationship for both preterm birth and pre‐eclampsia, whereby CVD risk is greater with the number of affected pregnancies,^60^, ^61^ the limited evaluation of a dose–response relationship for GH needs addressing.

## CONCLUSIONS

In conclusion, we found that GH is associated with a greater risk of overall CVD, specifically CHD and heart failure. The greater risk associated with many of these events is similar to other pregnancy complications, such as preterm birth and fetal growth restriction. Women who experience GH should be aware of this greater risk and may benefit from prenatal and postnatal counseling to increase their awareness of strategies that can reduce their CVD risk during and after birth.

## Sources of Funding

C.O.‐W. was supported by the British Heart Foundation Cambridge Centre of Excellence (RE/13/6/30180) and an early career fellowship from Homerton College, University of Cambridge. A.F. is supported by a UK Medical Research Council Fellowship (MR/M009351/1). The funding source had no role in the design or conduct of the study, collection or analysis of the data, or the decision to submit the manuscript for publication.

## Disclosures

None.

## Supporting information


**Tables S1–S10 Figures S1–S6 References 11–18, and 29–41**

## Notes

1 References 11, 12, 14, 16, 29, 30, 33, 34, 35, 37, 38, 39.

2 References 12, 13, 15, 16, 17, 29, 30, 32, 35, 41.
