# Supplementary material for: Future Cardiovascular Disease Risk for Women With Gestational Hypertension: A Systematic Review and Meta‐Analysis
Source: J Am Heart Assoc. 2020 Jun 24;9(13):e013991. doi: 10.1161/JAHA.119.013991 (PMC7670531; doi:10.1161/JAHA.119.013991)
Supplement: Supplementary file 1 — Tables S1–S10 Figures S1–S6 References 11–18, and 29–41 [file JAH3-9-e013991-s001.pdf]

Supplemental Material

Table S1. PRISMA checklist.

Section/topic	#	Checklist item	Reported on page #
**TITLE**			
Title	1	Identify the report as a systematic review, meta-analysis, or both.	1
**ABSTRACT**			
Structured summary	2	Provide a structured summary including, as applicable: background; objectives; data sources; study eligibility criteria, participants, and interventions; study appraisal and synthesis methods; results; limitations; conclusions and implications of key findings; systematic review registration number.	2
**INTRODUCTION**			
Rationale	3	Describe the rationale for the review in the context of what is already known.	3
Objectives	4	Provide an explicit statement of questions being addressed with reference to participants, interventions, comparisons, outcomes, and study design (PICOS).	3
**METHODS**			
Protocol and registration	5	Indicate if a review protocol exists, if and where it can be accessed (e.g., Web address), and, if available, provide registration information including registration number.	3
Eligibility criteria	6	Specify study characteristics (e.g., PICOS, length of follow-up) and report characteristics (e.g., years considered, language, publication status) used as criteria for eligibility, giving rationale.	3
Information sources	7	Describe all information sources (e.g., databases with dates of coverage, contact with study authors to identify additional studies) in the search and date last searched.	3
Search	8	Present full electronic search strategy for at least one database, including any limits used, such that it could be repeated.	Table S3
Study selection	9	State the process for selecting studies (i.e., screening, eligibility, included in systematic review, and, if applicable, included in the meta-analysis).	3-4
Data collection process	10	Describe method of data extraction from reports (e.g., piloted forms, independently, in duplicate) and any processes for obtaining and confirming data from investigators.	4
Data items	11	List and define all variables for which data were sought (e.g., PICOS, funding sources) and any assumptions and simplifications made.	4
Risk of bias in individual studies	12	Describe methods used for assessing risk of bias of individual studies (including specification of whether this was done at the study or outcome level), and how this information is to be used in any data synthesis.	4
Summary measures	13	State the principal summary measures (e.g., risk ratio, difference in means).	4

Synthesis of results	14	Describe the methods of handling data and combining results of studies, if done, including measures of consistency (e.g., I^2) for each meta-analysis.	4
Risk of bias across studies	15	Specify any assessment of risk of bias that may affect the cumulative evidence (e.g., publication bias, selective reporting within studies).	5
Additional analyses	16	Describe methods of additional analyses (e.g., sensitivity or subgroup analyses, meta-regression), if done, indicating which were pre-specified.	4-5
**RESULTS**			
Study selection	17	Give numbers of studies screened, assessed for eligibility, and included in the review, with reasons for exclusions at each stage, ideally with a flow diagram.	5, Fig 1
Study characteristics	18	For each study, present characteristics for which data were extracted (e.g., study size, PICOS, follow-up period) and provide the citations.	5, 9-13, Tables S4, S5 & S7
Risk of bias within studies	19	Present data on risk of bias of each study and, if available, any outcome level assessment (see item 12).	Tables S6 & S10
Results of individual studies	20	For all outcomes considered (benefits or harms), present, for each study: (a) simple summary data for each intervention group (b) effect estimates and confidence intervals, ideally with a forest plot.	Table S8 & S9, Fig 3 & 4 Fig S1, S2 & S3
Synthesis of results	21	Present results of each meta-analysis done, including confidence intervals and measures of consistency.	Fig 2, 3 & 4 Fig S1, S2 & S3
Risk of bias across studies	22	Present results of any assessment of risk of bias across studies (see Item 15).	5, 7,Table S11, Fig S4, S5 & S6
Additional analysis	23	Give results of additional analyses, if done (e.g., sensitivity or subgroup analyses, meta-regression [see Item 16]).	6-7, Table S9 & S10
**DISCUSSION**			
Summary of evidence	24	Summarize the main findings including the strength of evidence for each main outcome; consider their relevance to key groups (e.g., healthcare providers, users, and policy makers).	7-9
Limitations	25	Discuss limitations at study and outcome level (e.g., risk of bias), and at review-level (e.g., incomplete retrieval of identified research, reporting bias).	8
Conclusions	26	Provide a general interpretation of the results in the context of other evidence, and implications for future research.	7-9
**FUNDING**			
Funding	27	Describe sources of funding for the systematic review and other support (e.g., supply of data); role of funders for the systematic review.	1

Table S2. MOOSE Checklist for Meta-analyses of Observational Studies.

Item No	Recommendation	Reported on Page No
Reporting of background should include		
1	Problem definition	3
2	Hypothesis statement	3
3	Description of study outcome(s)	3
4	Type of exposure or intervention used	3
5	Type of study designs used	3
6	Study population	3
Reporting of search strategy should include		
7	Qualifications of searchers (eg, librarians and investigators)	4
8	Search strategy, including time period included in the synthesis and key words	3
9	Effort to include all available studies, including contact with authors	3
10	Databases and registries searched	3
11	Search software used, name and version, including special features used (eg, explosion)	3-4
12	Use of hand searching (eg, reference lists of obtained articles)	3
13	List of citations located and those excluded, including justification	Figure 1
14	Method of addressing articles published in languages other than English	None found
15	Method of handling abstracts and unpublished studies	None found
16	Description of any contact with authors	None required
Reporting of methods should include		
17	Description of relevance or appropriateness of studies assembled for assessing the hypothesis to be tested	5
18	Rationale for the selection and coding of data (eg, sound clinical principles or convenience)	4
19	Documentation of how data were classified and coded (eg, multiple raters, blinding and interrater reliability)	4
20	Assessment of confounding (eg, comparability of cases and controls in studies where appropriate)	4, Table S5
21	Assessment of study quality, including blinding of quality assessors, stratification or regression on possible predictors of study results	4, Table S4, TableS 9
22	Assessment of heterogeneity	4
23	Description of statistical methods (eg, complete description of fixed or random effects models, justification of whether the chosen models account for predictors of study results, dose-response models, or cumulative meta-analysis) in sufficient detail to be replicated	4
24	Provision of appropriate tables and graphics	Fig 1, Tables S1-S7
Reporting of results should include		
25	Graphic summarizing individual study estimates and overall estimate	Fig 2-4, Fig S1,S2
26	Table giving descriptive information for each study included	Table S4,S5,S7

27	Results of sensitivity testing (eg, subgroup analysis)	7, Table S10-S11, Fig S3
28	Indication of statistical uncertainty of findings	Fig 2-4, Fig S1, S2
Reporting of discussion should include		
29	Quantitative assessment of bias (eg, publication bias)	8, Fig S4-S6
30	Justification for exclusion (eg, exclusion of non-English language citations)	n/a
31	Assessment of quality of included studies	8, Table S6-S7
Reporting of conclusions should include		
32	Consideration of alternative explanations for observed results	8-9
33	Generalization of the conclusions (ie, appropriate for the data presented and within the domain of the literature review)	8
34	Guidelines for future research	9
35	Disclosure of funding source	1

Table S3. PubMed Search Strategy.

Population	("humans"[MeSH Terms] OR "Women"[Mesh] OR "Female"[Mesh] OR "Pregnancy"[Mesh]) AND
Exposure	("Hypertension, Pregnancy-Induced"[Mesh] OR “Gestational hypertension*”[All Fields] OR “Pregnancy Induced Hypertension*”[All Fields] OR “Transient Hypertension* in Pregnancy”[All Fields]) AND
Outcome	(“Acute Coronary Syndrome”[All Fields] OR “Aneurysm”[All Fields] OR “Angina”[All Fields] OR “Aortic Stenosis”[All Fields] OR “Aortic Stenoses”[All Fields] OR “Aortic Valve Insufficienc*”[All Fields] OR “Aortic Valve Stenosis”[All Fields] OR “Aortic Valve Stenoses”[All Fields] OR “Arrhythmia*”[All Fields] OR “Atrial Fibrillation*”[All Fields] OR “Atrial Flutter*”[All Fields] OR “Bradycardia”[All Fields] OR “Cardiac Arrest*”[All Fields] OR “Cardiac Oedema”[All Fields] OR “Cardiac edema”[All Fields] OR “Cardiac Tamponade”[All Fields] OR “Cardiomegal*”[All Fields] OR “Cardiomyopath*”[All Fields] OR “Cardiovascular Disease*”[All Fields] OR “CVD”[All Fields] OR “Cerebrovascular Disease*”[All Fields] OR “Cerebrovascular Disorder*”[All Fields] OR “Cerebral infarction*”[All Fields] OR “Cerebral haemorrhage*”[All Fields] OR “Cerebral hemorrhage*”[All Fields] OR “Commotio Cordis”[All Fields] OR “Coronary Artery Disease*”[All Fields] OR “Coronary Disease*”[All Fields] OR “CHD”[All Fields] OR “Coronary Occlusion*”[All Fields] OR “Coronary Restenosis”[All Fields] OR “Coronary Restenoses”[All Fields] OR “Coronary Stenosis”[All Fields] OR “Coronary Stenoses”[All Fields] OR “Coronary Vasospasm”[All Fields] OR “Emboli”[All Fields] OR “Embolism”[All Fields] OR “Endocarditis”[All Fields] OR “Heart Arrest*”[All Fields] OR “Heart Attack*”[All Fields] OR “Heart Block*”[All Fields] OR “Heart Disease*”[All Fields] OR “Heart Failure*”[All Fields] OR “Heart Rupture*”[All Fields] OR “Heart Valve Disease*”[All Fields] OR “Heart Valve Prolapse*”[All Fields] OR “Hypertroph*”[All Fields] OR “Intracranial Haemorrhage*”[All Fields] OR “Intracranial Hemorrhage*”[All Fields] OR “Long QT Syndrome”[All Fields] OR “Mitral Valve Insufficienc*”[All Fields] OR “Myocardial Infarction*”[All Fields] OR “Myocardial Ischemia”[All Fields] OR “Myocardial Ischaemia”[All Fields] OR “Myocardial Reperfusion Injury”[All Fields] OR “Myocardial Stunning”[All Fields] OR “Paroxysmal Dyspnea”[All Fields] OR “Peripheral arterial disease”[All Fields] OR “Pre-Excitation Syndrome”[All Fields] OR “Pulmonary Valve Insufficiency”[All Fields] OR “Pulmonary Valve Stenosis”[All Fields] OR “Pulmonary Valve Stenoses”[All Fields] OR “Pulmonary Heart Disease”[All Fields] OR “Stroke”[All Fields] OR “Sudden Cardiac”[All Fields] OR “Subarachnoid haemorrhage”[All Fields] OR “Subarachnoid hemorrhage”[All Fields] OR “Tachycardia”[All Fields] OR “Thrombosis”[All Fields] OR “Thromboses”[All Fields] OR “Transient Ischaemic Attack”[All Fields] OR “Transient Ischemic Attack”[All Fields] OR “Tricuspid Valve Insufficiency”[All Fields] OR “Tricuspid Valve Stenosis”[All Fields] OR “Tricuspid Valve Stenoses”[All Fields] OR “Ventricular Dysfunction”[All Fields] OR “Ventricular Fibrillation”[All Fields] OR “Ventricular Flutter”[All Fields] OR "Acute Coronary Syndrome"[Mesh] OR "Aneurysm"[Mesh] OR "Angina Pectoris"[Mesh] OR "Aortic Valve Stenosis"[Mesh] OR "Aortic Valve Insufficiency"[Mesh] OR "Arrhythmias, Cardiac"[Mesh] OR "Atrial Fibrillation"[Mesh] OR "Atrial Flutter"[Mesh] OR "Bradycardia"[Mesh] OR "Heart Arrest"[Mesh] OR "Edema, Cardiac"[Mesh] OR "Cardiac Tamponade"[Mesh] OR "Cardiomegaly"[Mesh] OR "Cardiomyopathies"[Mesh] OR "Cardiovascular Diseases"[Mesh] OR "Cerebrovascular Disorders"[Mesh] OR "Commotio Cordis"[Mesh]) OR "Coronary Artery Disease"[Mesh] OR "Coronary Disease"[Mesh] OR "Coronary Occlusion"[Mesh] OR "Coronary Restenosis"[Mesh] OR "Coronary Stenosis"[Mesh] OR "Coronary Vasospasm"[Mesh] OR "Embolism"[Mesh] OR "Endocarditis"[Mesh] OR "Myocardial Infarction"[Mesh] OR "Heart Block"[Mesh] OR "Heart Diseases"[Mesh] OR "Heart Failure"[Mesh] OR "Heart Rupture"[Mesh] OR "Heart Valve Diseases"[Mesh] OR "Heart Valve Prolapse"[Mesh] OR "Hypertrophy"[Mesh] OR "Intracranial Hemorrhages"[Mesh] OR "Long QT Syndrome"[Mesh] OR "Mitral Valve Insufficiency"[Mesh] OR "Myocardial Ischemia"[Mesh] OR "Myocardial Reperfusion Injury"[Mesh] OR "Myocardial Stunning"[Mesh] OR "Dyspnea, Paroxysmal"[Mesh] OR "Peripheral Arterial Disease"[Mesh] OR "Pre-Excitation Syndromes"[Mesh] OR "Pulmonary Valve Insufficiency"[Mesh] OR "Pulmonary Valve Stenosis"[Mesh] OR "Pulmonary Heart Disease"[Mesh] OR "Stroke"[Mesh] OR "Death, Sudden, Cardiac"[Mesh] OR "Subarachnoid Hemorrhage"[Mesh] OR "Tachycardia"[Mesh] OR "Thrombosis"[Mesh] OR "Ischemic Attack, Transient"[Mesh] OR "Tricuspid Valve Insufficiency"[Mesh] OR "Tricuspid Valve Stenosis"[Mesh] OR "Ventricular Dysfunction"[Mesh] OR "Ventricular Fibrillation"[Mesh] OR "Ventricular Flutter"[Mesh]) AND
Study Design	(“longitudinal studies"[MeSH Terms] OR “longitudinal study”[All Fields] OR “longitudinal studies”[All Fields] OR "prospective"[All Fields] OR “cohort”[All Fields] OR “cohorts”[All Fields] OR “follow up”[All Fields] OR “follow-up”[All Fields] OR "Epidemiology"[Mesh] OR "Epidemiology"[All Fields] OR "Epidemiological"[All Fields] OR "Retrospective Studies"[Mesh] OR "Retrospective"[All Fields] OR “prospective”[All Fields] OR "Cross-Sectional Studies"[Mesh] OR "Cross-Sectional"[All fields] OR "Cross Sectional"[All fields] OR "Case-Control Studies"[Mesh] OR "Case-Control"[All Fields])

Table S4. Definitions of Cardiovascular Events.

First author, year	Definition
Andolf et al. 2017[30]	Coronary Heart Disease: ICD-10 (I20-25)<br>Stroke: ICD-10 (I60-69)<br>Heart Failure: ICD-10 (I50)
Behrens et al. 2016[31]	Congestive Heart Failure: ICD-8 (427.09-427.19, 427.99, 428.99, 782.49); ICD-10 (I50.0-50.9);<br>Cardiomyopathy: ICD-8 (425.99); ICD-10 (I42.0-43.8, O90.3)
Bhattacharya et al. 2012[11]	CHD: ICD-9 (410-4, 428); ICD-10 (I20-5, I50);<br>Stroke: ICD-9 (430-8); ICD-10 (I60-9);<br>CVD: ICD-9 (390-459); ICD-10 (I00-I99, G45)
Cain et al. 2016[32]	CVD: ICD-9 codes for CHD, cerebrovascular disease, peripheral artery disease, or congestive heart failure, or for cardiac or peripheral arterial revascularization that were not specified
Cirillo et al. 2015[33]	CVD mortality: ICD-7 (420.1); ICD-8 (410, 412); ICD-9 (410, 411, 414, 429), ICD-10 (I21, I24, I25)
Grandi et al. 2017[14]	CVD: Read codes for cerebrovascular disease, CHD, coronary revascularization, MI, peripheral arterial disease, transient ischaemic attack and stroke
Kestenbaum et al. 2003[15]	Thromboembolism: ICD-9 (451.1, 453, 415.1);<br>CVD: ICD-9 (410, 430, 431, 434, 436), coronary artery revascularization procedure, including coronary artery bypass grafting (procedure code:36)
Lin et al. 2016[29]	Intracerebral haemorrhage: ICD-9 (430–432)
Luoto et al. 2008[12]	CVD: ICD-9 (389-459); ICD-10 (I00-I99)
Lykke et al. 2009[35]	CHD: ICD-8 (410-414), ICD-10 (I20-I25);<br>Heart Failure: ICD-8 (42709-42711, 42719, 42799, 42899, 42900, 42908, 42909), ICD-10 (I50, I51.3, I51.9)<br>Thromboembolic event: ICD-8 (444, 450-1), ICD-10 (I26, I74, I82)<br>Stroke: ICD-8 (430-438), ICD-10 (I60-I67, G45)
Lykke et al. 2010[34]	CVD: ICD-8 (39-44, 451-458), ICD-10 (DI0-DI9)
Männistö et al. 2013[36]	CHD, MI, Heart failure, Ischemic stroke: ICD codes, which were not specified
Ray et al. 2005[37]	CVD: ICD-9, ICD-10 codes, which were not specified
Riise et al. 2018[38]	CVD: ICD-9 (390–459); ICD-10 (I00–I99, except I84);<br>CHD: ICD-9 (410–414); ICD-10 (I20–I25);<br>Stroke: ICD-9 (430–438); ICD-10 (I60–I69)
Riise et al. 2019[39]	Acute MI or acute cerebral stroke - composite of hospitalization with AMI: ICD-9 (410); ICD-10 (I21-22); death from CHD: ICD-9 (410-414), ICD-10 (I20-25); hospitalization or death with acute cerebral stroke: ICD-9 (43), ICD-10 (I60-61, I63-64, except I63.6)

Schmiegelow et al. 2014[16]	MI: ICD-10 (I21-I22);<br>CVD: ICD-10 (I00-I99);<br>Ischemic stroke: ICD-10 (I63-I64).
Theilen et al. 2016[17]	CVD: ICD-9 (390–459);<br>CHD, Stroke: Codes not specified
Tooher et al. 2017[13]	CHD, Stroke: ICD-9 & ICD-10 codes, which weren't specified
Wikstrom et al. 2005[40]	CHD: ICD-9 (410–414), ICD-10 (I20–I25)
Wilson et al 2003[41]	Angina, MI, DVT: ascertained through the women's general practitioner, medical and death records<br>Other circulatory disease: ICD-9 (390-8, 405, 415-27, 440-59), ICD-10 (I00-9, I15, I26-8, I30-49, I51-2, I70-99)
Yeh et al. 2014[18]	CVD, ICD-9 (390-459)

CHD – coronary heart disease; CVD – cardiovascular disease; ICD – International classification of diseases; MI – myocardial infarction

Table S5. Risk of Bias Assessment in Prospective Studies.

First author, year	Selection	Comparability	Outcome	Overall Assessment
Andolf et al. 2017 [30]	★★★	★★	★★	Low Risk of Bias
Behrens et al. 2016[31]	★★★★	★★	★★★	Low Risk of Bias
Bhattacharya et al. 2012[11]	★★★	★★	★★	Low Risk of Bias
Cain et al. 2016[32]	★★★★	★★	★★	Low Risk of Bias
Cirillo et al. 2015[33]	★★★★	★★	★★★	Low Risk of Bias
Grandi et al. 2017[14]	★★★★	★★	★	High Risk of Bias
Kestenbaum et al. 2003 [15]	★★★★	★★	★	High Risk of Bias
Lin et al. 2016[29]	★★★★	★	★	High Risk of Bias
Luoto et al. 2008[12]	★★	★★	★ ★	Moderate Risk of Bias
Lykke et al. 2009[35]	★★★★	★★	★★	Low Risk of Bias
Lykke et al. 2010[34]	★★★★	★★	★★	Low Risk of Bias
Männistö et al. 2013[36]	★★★	★★	★★	Low Risk of Bias
Ray et al. 2005[37]	★★★★	★★	★★	Low Risk of Bias
Riise et al. 2018[38]	★★★★	★★	★★★	Low Risk of Bias
Riise et al. 2019[39]	★★★★	★★	★★★	Low Risk of Bias
Schmiegelow et al. 2014[16]	★★★★	★★	★★	Low Risk of Bias
Theilen et al. 2016[17]	★★★★	★★	★	High Risk of Bias
Tooher et al. 2017[13]	★★★	★★	★	High Risk of Bias
Wikstrom et al. 2005[40]	★★★★	★★	★★	Low Risk of Bias
Wilson et al. 2003[41]	★★★	★★	★★	Low Risk of Bias
Yeh et al. 2014[18]	★★★★	★	★★	Low Risk of Bias

Acceptable loss of follow-up taken to be <10%; Sufficient duration of follow-up taken to be from average age at pregnancy to after menopause (52 years old)

Table S6. Adjustments of Included Studies.

First author, year	Adjustment factors	Quality of adjustment
Andolf et al. 2017[30]	Mother's age at birth, mother's attained educational level in 1985, marital status and origin (Nordic/non-Nordic), history of cardiovascular disease later in life (diabetes, arteriosclerosis, stroke, ischemic heart disease, heart failure and hypertension)	Adequate
Behrens et al. 2016[31]	Maternal age, maternal birth year, parity, multiple pregnancy and stillbirth	Poor
Bhattacharya et al. 2012[11]	Year of birth, social class and smoking	Poor
Cain et al. 2016[32]	Age, race/ethnicity, nativity, education, income, 5-year history of hyperlipidemia, migraine, lupus; pre-pregnancy BMI, gestational diabetes, tobacco use, drug use, and infant sex	Well
Cirillo et al. 2015[33]	Age, race, parity, BMI, and cigarette smoking	Well
Grandi et al. 2017[14]	Age, smoking, BMI, excessive alcohol use, year of cohort entry, region of residence, multiple gestation at first pregnancy, depression, dyslipidaemia, venous thromboembolism, polycystic ovary syndrome, gestational diabetes (measured between 12 weeks of gestation and 6 weeks post-partum), diabetes mellitus, renal disease, migraines, family history of hypertension and family history of cardiovascular disease any time before cohort entry, number of distinct drug classes prescribed, and use of statin, aspirin and anti-depressant medications in the year prior to pregnancy	Well
Kestenbaum et al. 2003[15]	Age, parity, calendar year of delivery	Poor
Lin et al. 2016[29]	Age, follow-up years	Poor
Luoto et al. 2008[12]	Age, hormone use, height, marital status and visit to private doctor	Adequate
Lykke et al. 2009[35]	Age, year of delivery, preterm delivery, SGA offspring, placental abruption, stillbirth and later type 2 diabetes mellitus	Adequate
Lykke et al. 2010[34]	Age, year of delivery.	Poor
Männistö et al. 2013[36]	Age at pregnancy, pre-pregnancy BMI, smoking, parity, diabetes mellitus before/during pregnancy, socioeconomic status	Well
Ray et al. 2005[37]	Age, multiple gestation, length of stay, income quintile, rural residence, drug dependence, and gestational diabetes mellitus in index delivery, and hypertension, any diabetes mellitus, obesity, dyslipidaemia, tobacco use, renal disease, migraine headache, and systemic lupus erythematosus	Well
Riise et al. 2018[38]	Age, educational level, marital status, and birth year of first child	Poor
Riise et al. 2019[39]	Age at recruitment age at first delivery, education (primary, high school/vocational, any college/ university) and a family history of MI prior to age 60	Well
Schmiegelow et al. 2014[16]	Age, smoking, and year of inclusion	Poor
Theilen et al. 2016[17]	Age, year of childbirth, parity, infant sex, parental education, preterm delivery, race-ethnicity, maternal marital status	Adequate
Tooher et al. 2017[13]	Age, gestation, and parity	Poor

Wikstrom et al. 2005[40]	Age, socio-economic level and category of hospital	Poor
Wilson et al. 2003[41] *	Age, BMI, social class, and smoking habit.	Adequate
Yeh et al. 2014[18]	Age, diabetes, dyslipidemia, incident hypertension, date of delivery	Poor

* Risk estimates for “other circulatory disease” were adjusted for age at delivery and social class only, and is considered poorly adjusted

Table S7. Results of Studies Included in the Meta-analysis by Outcome.

Outcome	First author, year	Exposure definition	Cases (N)	Point Estimate	Unadjusted or Age-adjusted Results	Adjusted Results *
Cardiovascular Disease	Bhattacharya et al. 2012[11]	GH in 1st pregnancy	1,319	IRR	1.19 (1.06,1.34)	1.25 (1.11,1.41)
	Cain et al. 2016[32]	GH in 1st pregnancy	2447	HR	1.18 (1.01, 1.37)	0.99 (0.85, 1.16)
	Grandi et al. 2017[14]	GH in 1st pregnancy	920 †	HR	2.4 (1.9, 3.1)	2.3 (1.8, 2.9)
	Luoto et al. 2008[12]	GH in 1st pregnancy	38	HR	0.87 (0.61, 1.25)	0.90 (0.62, 1.30)
	Lykke et al. 2010[34]	GH in 1st pregnancy	1,194	HR	NG	2.47 (1.74, 3.52)
	Ray et al. 2005[37]	GH in 1st pregnancy	1,987	HR	NG	1.8 (1.4, 2.2)
	Riise et al. 2018[38]	GH in 1st pregnancy	19,869	HR	1.8 (1.7, 2.0)	1.8 (1.7, 2.0)
	Cirillo et al. 2015 ‡[33]	A history of GH	9,000 †	HR	African American: 1.70 (1.10, 2.65)<br>non-African American: 0.90 (0.63,1.36)	African American: 1.8 (1.09, 2.82)<br>non-African American: 1.0 (0.68, 1.52)
	Kestenbaum et al. 2003[15]	A history of GH	83	HR	2.9 (1.8, 4.9)	2.8 (1.6, 4.8)
	Luoto et al. 2008[12] *	A history of GH	98	HR	1.18 (0.99, 1.40)	1.17 (0.98, 1.41)
	Schmiegelow et al. 2014[16]	A history of GH	374	HR	NG	2.77 (1.47, 5.21)
	Theilen et al. 2016[17]	A history of GH	NG	HR	NG	2.39 (1.78, 3.21)
	Yeh et al. 2014[18]	A history of GH	182	HR	NG	2.00 (1.26, 3.18)
Coronary Heart Disease	Bhattacharya et al. 2012[11]	GH in 1st pregnancy	681	IRR	1.09 (1.00,1.19)	1.22 (1.11, 1.34)
	Lykke et al.2009[35]	GH in 1st pregnancy	2,271	HR	1.67 (1.41, 1.97)	1.48 (1.25, 1.76)
	Riise et al. 2018[38]	GH in 1st pregnancy	2,364	HR	1.7 (1.3, 2.2)	1.7 (1.3, 2.1)
	Wikstrom et al. 2005[40]	GH in 1st pregnancy	2,142	IRR	2.0 (1.7, 2.5)	1.6 (1.3, 2.0)
	Andolf et al. 2017 [30] §	A history of GH	10,755 †	HR	1.33 (1.20, 1.48)	1.26 (1.13, 1.40)
	Männistö et al. 2013[36]	A history of GH	1,225	HR	NG	1.44 (1.24, 1.68)
	Tooher et al. 2017[13] §	A history of GH	NG	OR	NG	3.19 (2.11, 4.83)
	Theilen et al. 2016[17]	A history of GH	NG	HR	NG	2.77 (1.62, 4.75)
Stroke ‖	Bhattacharya et al. 2012[11]	GH in 1st pregnancy	2,638	IRR	0.97 (0.86,1.09)	1.04 (0.91,1.18)
	Lykke et al. 2009[35]	GH in 1st pregnancy	8,987	HR	1.68 (1.42, 1.97)	1.51 (1.26, 1.81)
	Riise et al. 2018[38]	GH in 1st pregnancy	2,452	HR	1.3 (0.9, 1.7)	1.3 (0.9, 1.7)
	Andolf et al. 2017 [30] §	A history of GH	7,436 †	HR	1.36 (1.20, 1.55)	1.30 (1.14, 1.48)
	Tooher et al. 2017[13] §	A history of GH	NG	OR	NG	0.57 (0.14, 2.31)
	Theilen et al. 2016[17]	A history of GH	NG	HR	NG	2.97 (1.49, 5.92)
Heart Failure	Andolf et al. 2017 [30]	A history of GH	3,165 †	HR	1.62 (1.36, 1.93)	1.52 (1.28, 1.80)
	Männistö et al. 2013[36]	A history of GH	486	IRR	NG	1.79 (1.43, 2.21)
	Behrens et al. 2016[31]	A history of GH	3,581	HR	NG	2.07 (1.70, 2.52)

Thromboembolic events¶	Bhattacharya et al. 2012[11]	GH in 1st pregnancy	384	IRR	0.82 (0.65,1.04)	0.86 (0.67,1.09)
	Lykke et al. 2009[35]	GH in 1st pregnancy	3,881	HR	1.01 (0.72-1.40)	1.03 (0.73, 1.45)
	Wilson et al. 2003[41]	GH in 1st pregnancy	47	OR	NG	0.65 (0.35, 1.20)

GH – gestational hypertension; NG – not given; HR – Hazard Ratio, OR – Odds Ratio, IRR – incident rate ratio

* See Table S4 for adjustment; † estimated; ‡ Results were combined by fixed effect meta-analysis to provide an estimate of the CVD risk for the whole population. § CHD and stroke results for each paper were combined by fixed effect meta-analysis to provide an estimate of the risk of CVD. ‖ Studies that reported all-cause stroke only. ¶ Study specific outcomes were: Wilson – Deep Vein Thrombosis, Bhattacharya - Pulmonary Embolism; Lykke – Thromboembolic Events

Table S8. Results of Studies Not Included in the Meta-analysis by Outcome.

Outcome	First author, year	Exposure definition	Cases (N)	Point Estimate	Unadjusted Results	Adjusted Results *
Heart Failure	Lykke et al 2009[35]	GH in 1st pregnancy	7,483	HR	1.57 (1.12-2.20)	1.37 (0.98-1.93)
Angina	Wilson et al. 2003[41]	GH in 1st pregnancy	64	OR	NG	1.02 (0.58 to 1.81)
Acute MI and acute cerebral stroke	Riise et al. 2019[39]	GH in 1st pregnancy	134	HR	2.4 (1.1-5.5)	1.8 (0.8-4.1)
Other circulatory disease †	Wilson et al. 2003[41]	GH in 1st pregnancy	172	IRR	NG	1.51 (1.06-2.14)
Myocardial Infarction	Wilson et al. 2003[41]	GH in 1st pregnancy	30	OR	NG	0.73 (0.32-1.63)
	Männistö et al. 2013[36]	A history of GH	471	IRR	NG	1.75 (1.40–2.19)
	Schmiegelow et al. 2014[16]	A history of GH	68	HR	NG	1.41 (0.19-10.21)
Intracerebral haemorrhage	Lin et al. 2016[29]	A history of GH	27	IRR	NG	3.72 (3.63-3.81)
Ischaemic Stroke	Männistö et al. 2013[36]	A history of GH	384	IRR	NG	1.59 (1.24-2.04)
	Schmiegelow et al. 2014[16]	A history of GH	175	HR	NG	2.78 (1.13-6.82)
Cardiomyopathy	Behrens et al. 2016[31]	A history of GH	1,448	HR	NG	1.83 (1.20-2.63)
Thromboembolic event	Kestenbaum et al. 2003[15]	A history of GH	127	HR	1.4 (0.8-2.4)	1.5 (0.9-2.5)
Cardiovascular Disease	Riise et al. 2018[38]	Pregnancies with GH in women with 2+ pregnancies	19,869	HR	NG	GH 1st pregnancy: 1.7 (1.5–2.0)<br>GH 2nd pregnancy: 2.4 (2.1–2.8)<br>2+ GH pregnancies: 1.9 (1.8–2.0)
Coronary Heart Disease	Wikstrom et al. 2005[40]	Pregnancies with GH in women with 2+ pregnancies	1,242	IRR	GH 1st pregnancy: 1.9 (1.5-2.4)<br>GH 2nd pregnancy: 2.7 (2.0–3.5)<br>2+ GH pregnancies: 3.3 (2.4–4.5)	GH 1st pregnancy: 1.9 (1.5-2.4)<br>GH 2nd pregnancy: 2.4 (1.8–3.2)<br>2+ GH pregnancies 2.8 (2.0–3.9)

GH – gestational hypertension; MI – myocardial infarction; NG – not given; HR – Hazard Ratio, OR – Odds Ratio, IRR – incident rate ratio
* See Table S4 for adjustment. † Other circulatory disease excluding hypertension, cerebrovascular disease or coronary heart disease

Table S9. Sensitivity Analyses of Risk of Cardiovascular Events Estimated from the Adjusted Meta-Analyses.

Outcome	Exposure	Sensitivity Analysis	Excluded Studies	RR	(95% CI)	I^2	(95% CI)
Cardiovascular Disease	GH in 1st pregnancy	Excluding study(s) with the largest effect	Grandi 2017[14]	1.35	(1.08-1.69)	92%	(86-96%)
		Fixed effects model	n/a	1.52	(1.44-1.61)	92%	(87-96%)
	A history of GH	Excluding study(s) with the largest effect	Kestenbaum 2003[15]; Schmiegelow 2014[16]	1.65	(1.28-2.11)	76%	(46-89%)
		Fixed effects model	n/a	1.39	(1.29-1.49)	85%	(70-93%)
Coronary Heart Disease	GH in 1st pregnancy	Excluding study(s) with the largest effect	Riise 2018[38]	1.40	(1.17-1.66)	73%	(10-92%)
		Fixed effects model	n/a	1.35	(1.25-1.45)	74%	(27-91%)
	A history of GH	Excluding study(s) with the largest effect	Tooher et al. 2017[13]	1.49	(1.18-1.89)	78%	(31-93%)
		Fixed effects model	n/a	1.39	(1.28-1.52)	88%	(72-95%)
Stroke	GH in 1st pregnancy	Excluding study(s) with the largest effect	Not conducted *	-	-	-	-
		Fixed effects model	n/a	1.19	(1.06-1.32)	82%	(44-94%)
	A history of GH	Excluding study(s) with the largest effect	Not conducted *	-	-	-	-
		Fixed effects model	n/a	1.33	(1.17-1.51)	70%	(0-91%)
Heart Failure	A history of GH	Excluding study(s) with the largest effect	Not conducted *	-	-	-	-
		Fixed effects model	n/a	1.75	(1.57-1.95)	63%	(0-90%)

CI – Confidence Intervals; GH – Gestational Hypertension; RR – Relative Risk

* Fewer than four studies included in meta-analysis, so sensitivity analysis was not conducted

Table S10. Stratified Analyses of the Risk of Cardiovascular Disease Estimated from the Adjusted Meta-analyses.

Exposure	Strata		Studies (N)	RR	(95% CI)	I^2	(95% CI)	P-value
GH in 1st pregnancy	Level of Adjustment	Adequately/Well	5	1.38	(1.26-1.52)	91%	(83-96%)	0.796
		Poor	2	1.60	(1.50-1.72)	82%	(53-93%)	
	Risk of Bias	Low Risk	5	1.51	(1.42-1.60)	93%	(87-96%)	0.904*
		Not Low Risk	2	1.75	(1.43-2.14)	94%	(82-98%)	
	Average follow-up	<20 years	4	1.63	(1.53-1.74)	93%	(86-96%)	0.281
		>20 years	3	1.21	(1.08-1.36)	63%	(0-92%)	
	Year of Publication	Up to 2010	3	1.50	(1.32-1.71)	80%	(35-94%)	0.781
		2010 onwards	4	1.53	(1.44-1.62)	96%	(92-98%)	
	Population	European	5	1.61	(1.51-1.71)	91%	(81-95%)	0.694*
		Non-European	2	1.20	(1.06-1.36)	95%	(83-98%)	
A history of GH	Level of Adjustment	Adequately/Well	3	1.34	(1.24-1.46)	87%	(64-96%)	0.417
		Poor	4	1.41	(1.21-1.65)	82%	(53-93%)	
	Risk of Bias	Low Risk	4	1.31	(1.21-1.43)	66%	(1-88%)	0.656*
		Not Low Risk	4	1.50	(1.29-1.74)	91%	(76-96%)	
	Average follow-up	<20 years	3	2.40	(1.77-3.27)	0%	(0-90%)	0.475
		>20 years	5	1.31	(1.22-1.42)	83%	(57-93%)	
	Year of Publication	Up to 2010	3	1.28	(1.08-1.52)	89%	(56-97%)	0.863
		2010 onwards	5	1.37	(1.27-1.48)	83%	(61-93%)	
	Population	European	3	1.27	(1.18-1.38)	70%	(0-91%)	0.303*
		Non-European	5	1.90	(1.58-2.28)	72%	(20-90%)	

CI – Confidence Intervals; GH – Gestational Hypertension; RR – Relative Risk; N - Number

* Test for interaction, all other – values are test for trend from meta-regression

Figure S1. Association between gestational hypertension in a woman's first pregnancy and subsequent risk of cardiovascular events in unadjusted analyses.

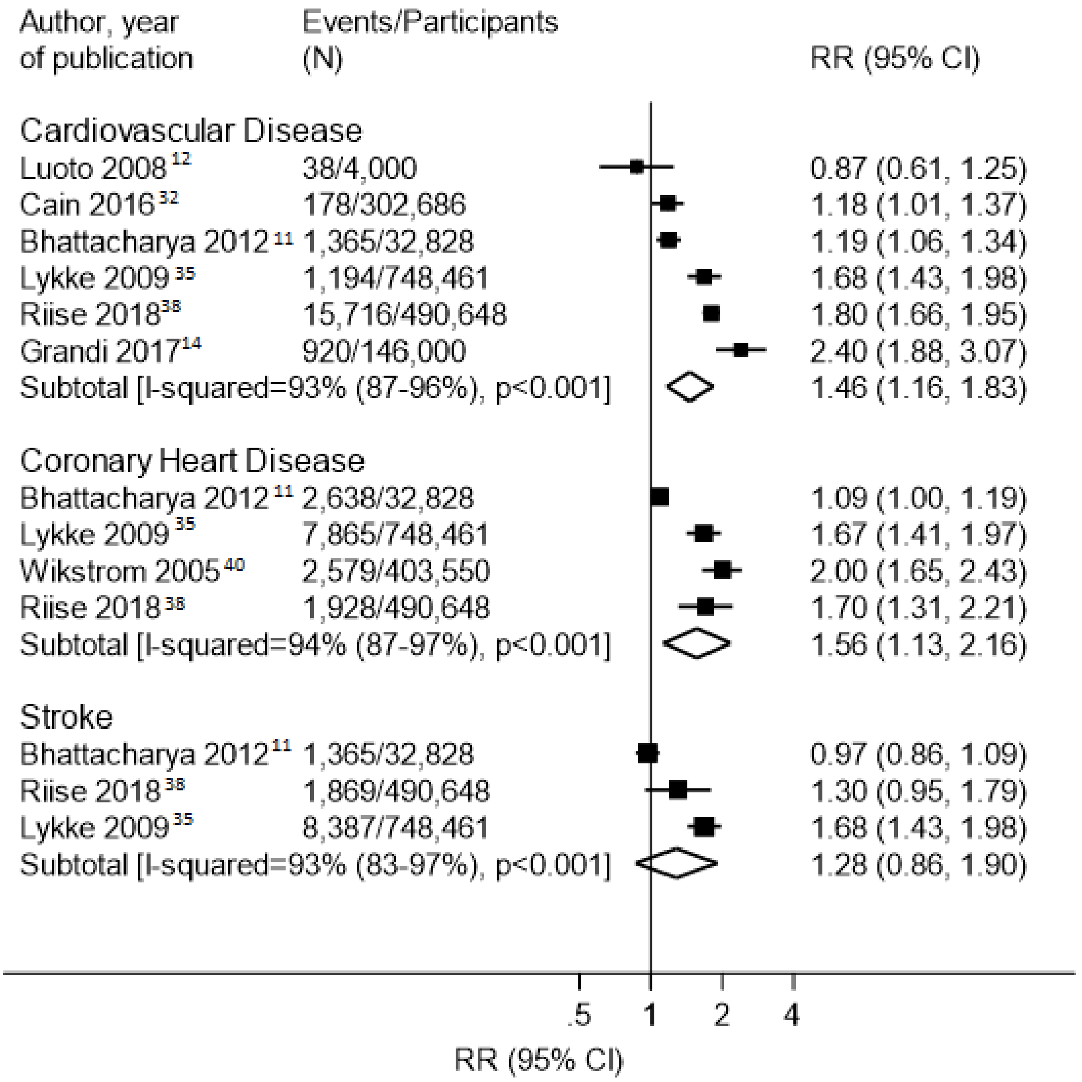

CI – Confidence intervals; RR – Relative Risk

Figure S2. Association between a history of one or more pregnancies affected by gestational hypertension and subsequent risk of cardiovascular events in unadjusted analyses.

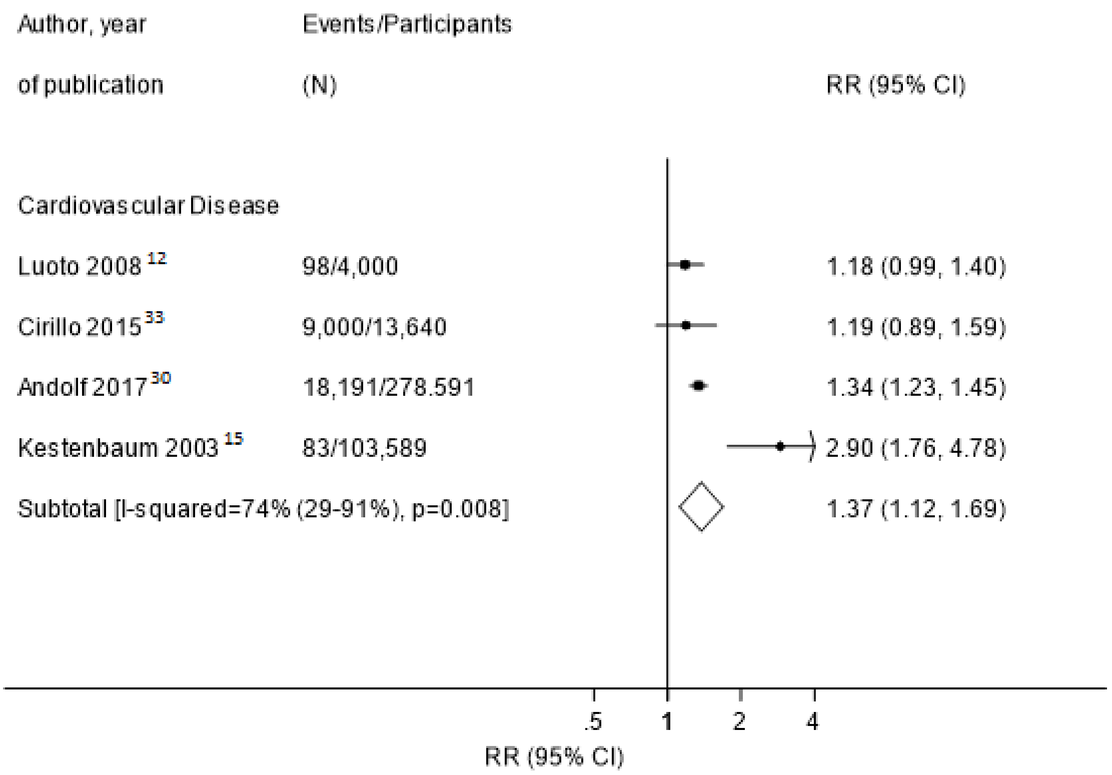

CI – Confidence intervals; RR – Relative Risk

Figure S3. Association between a history of one or more pregnancies affected by gestational hypertension and subsequent risk of any stroke event in adjusted analyses.

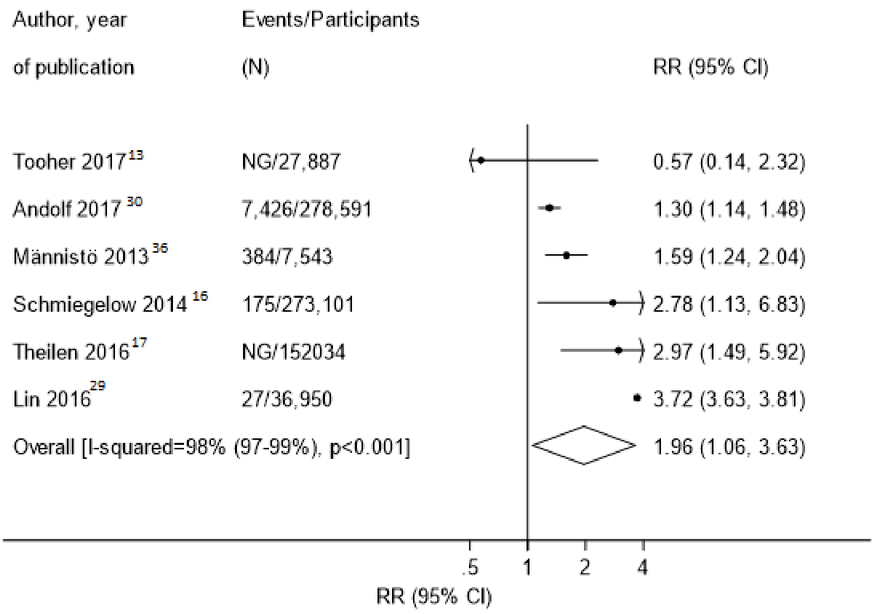

CI – Confidence intervals; NG – not given; RR – Relative Risk

Figure S4. Funnel plot of the studies contributing to the meta-analysis of the risk of cardiovascular disease after gestational hypertension in the first pregnancy.

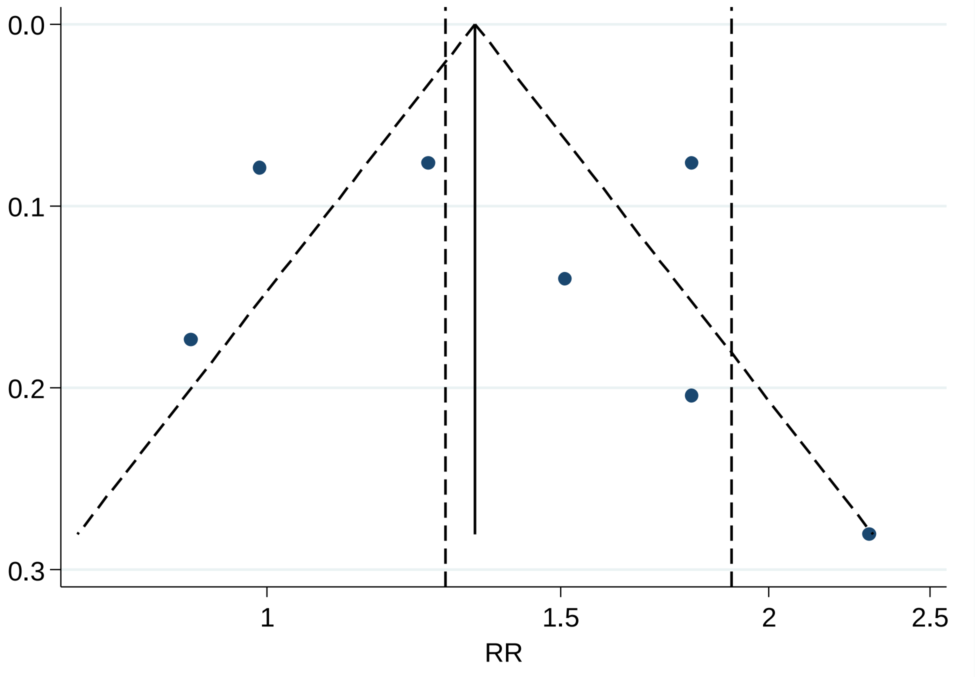

Egger's test p-value: 0.682. Vertical dashed lines indicate the confidence intervals from the pooled analysis. RR- Relative Risk

Figure S5. Funnel plot of the studies contributing to the meta-analysis of cardiovascular disease risk after a history of one or more pregnancies affected by gestational hypertension.

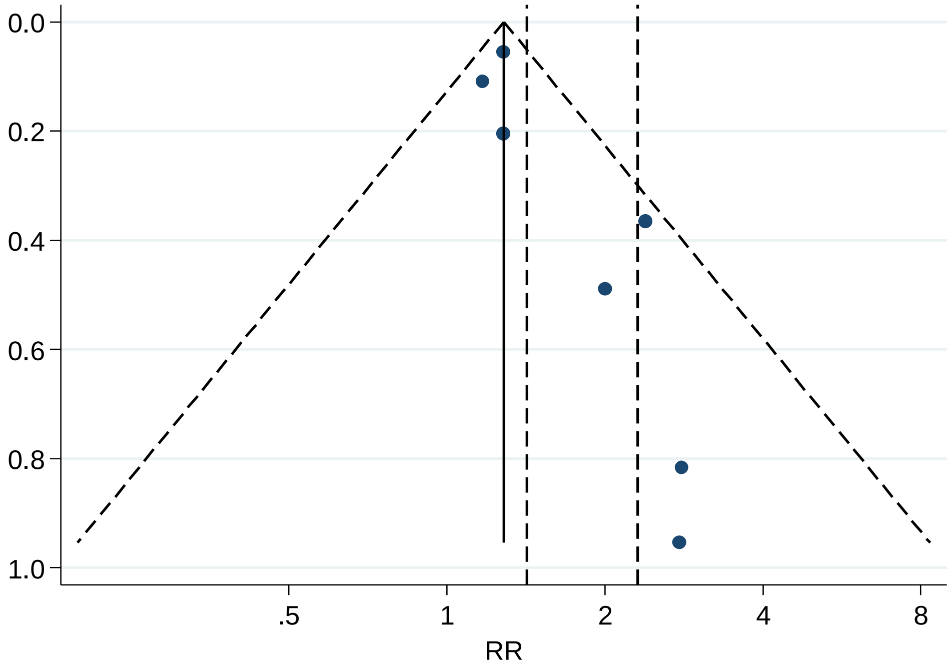

Egger's test p-value: 0.051. Trim-and-fill estimate: RR=1.26 (1.15-1.39). Vertical dashed lines indicate the confidence intervals from the pooled analysis. RR- Relative Risk

Figure S6. Funnel plot of the studies contributing to the meta-analysis the risk of any stroke event after a history of one or more pregnancies affected by gestational hypertension.

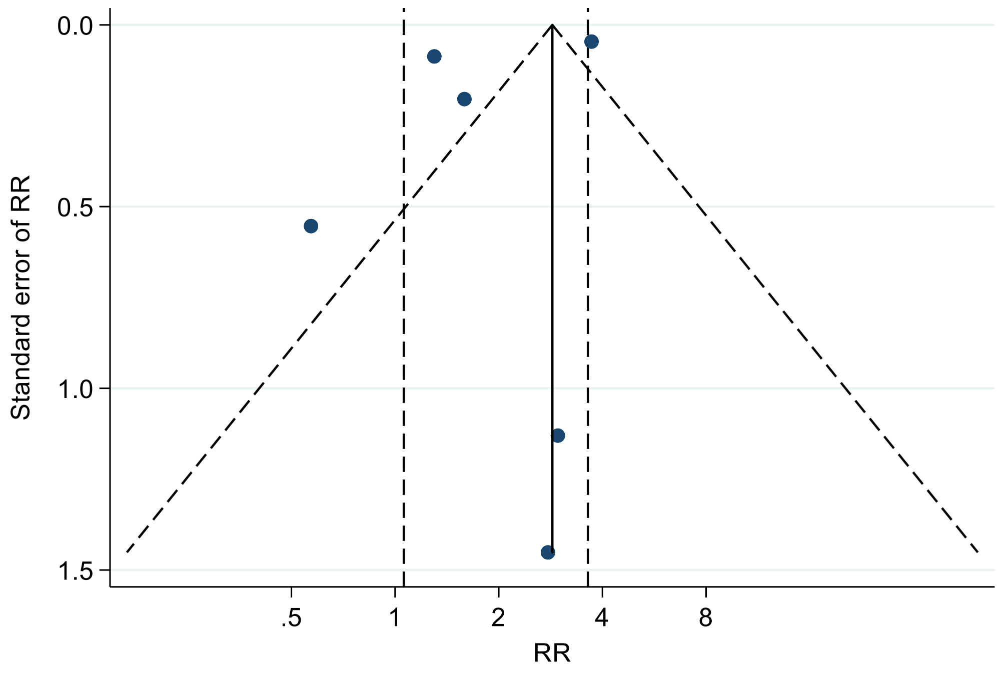

Egger's test p-value: 0.382. Vertical dashed lines indicate the confidence intervals from the pooled analysis. RR- Relative Risk
